# A Longitudinal Exploration of How Connections to Staff Facilitate Efficacy and Service Use in Drop-in Centers Serving Youth Experiencing Homelessness

**DOI:** 10.1007/s10935-023-00728-0

**Published:** 2023-03-16

**Authors:** Eric R. Rice, Graham DiGuiseppi, Laura Onasch-Vera, Erin Casey, Toni Cooper, Mischa DiBattiste

**Affiliations:** 1 Suzanne Dworak-Peck School of Social Work, University of Southern California, Los Angeles, CA 90015 USA; 2 My Friend’s Place, 5850 Hollywood Blvd, Los Angeles, CA 90028 USA; 3 Safe Place for Youth, 2469 Lincoln Blvd, Venice, CA 90291 USA

**Keywords:** Youth experiencing homelessness, Service use, Mediation, Knowledge, Self-efficacy, Drop-in center

## Abstract

**Introduction**: Youth experiencing homelessness (YEH) benefit from a variety of services to meet their immediate and long-term needs. Drop-in centers are a popular service venue used by YEH. However, the mechanisms responsible for engaging youth in drop-in services are not clear. The current study uses longitudinal data to explore the role of positive staff relationships in increasing youths’ knowledge and efficacy to access and subsequently use drop-in center services. **Methods**: 731 youth (*M*_age_ = 21.8, *SD =* 2.2, 25.1% female) accessing services at three drop-in centers in Los Angeles, California participated in the study. Surveys were completed at baseline, 1-month, and 3-months later. Path models examined the direct effect of positive relationships with adult staff on service use at the 3-month follow-up, and the indirect effect of service knowledge (assessed at the 1-month follow-up). **Results**: The direct effect model showed that positive staff relationships at baseline were significantly associated with number of services used at the 3-month follow-up (aIRR = 1.24, 95% CI: 1.00, 1.54). Positive staff relationships were also associated with greater service knowledge at 1-month (*b* = 0.93, *p* < 0.001), which in turn was associated with greater service use at 3-months (IRR = 1.15, 95% CI: 1.04, 1.28). The indirect effect of service knowledge was significant (*b* = 0.13, *p* = 0.02), suggesting that the association between positive staff relationships and service use was completely mediated by service knowledge. **Conclusions**: The current study adds to the literature by demonstrating that positive relationships with staff lead to increased service use by increasing youths’ knowledge and efficacy to access services. Efforts should be made to develop positive relationships with YEH in order to engage them in essential services needed to exit homelessness.

Recent data suggest that there are more than 4 million adolescents and young adult who experience homelessness in the United States each year (Morton et al., 2018). These youth experiencing homelessness (YEH) are a vulnerable population in need of a range of services to address both immediate (e.g., food, clothing, emergency shelter) and long-term needs (e.g. education, career, health long term housing solutions) (Gwadz et al., 2018; Pedersen et al., 2016). Drop-in centers, as points of immediate service needs, particularly food, clothing and connections to other social services are service centers typically preferred by youth over emergency or crisis shelters (Slesnick et al., 2016). Probability-based samples of youth experiencing homelessness sampled from street settings suggest that about one in six youth experiencing homelessness have accessed drop-in services recently in the past 30 days (Tucker et al., 2018). Drop-in services may also be more effective at reducing high-risk behaviors among youth. One study found that when randomly assigned to be referred to drop-in services or shelters services, youth who were referred to drop-in services evidenced significantly greater reductions in substance use and HIV risk behaviors lasting up to 9-months later (Slesnick et al., 2016). However, other studies suggest that youth are more likely to use drop-in centers for basic services (i.e., food, clothing, showers and hygiene products), rather than higher-level services such as case management (Kort-Butler & Tyler, 2012; Parast et al., 2019).

Given the high potential for drop-in centers to provide much needed services to YEH, one priority research area is to identify factors that facilitate drop-in service utilization. A recent review of the literature used the Gelberg-Andersen Behavioral Model for Vulnerable Populations (Gelberg et al., 2000) as a framework, and identified predisposing, enabling, and need factors associated with drop-in center service use (Pedersen et al., 2016). Among the enabling factors, positive relationships with adult staff emerged as a consistent predictor of drop-in center use. In a subsequent study among youth who utilized a drop-in center in the past 30 days in Los Angeles, youths’ reports of supportive drop-in center staff emerged as one significant enabling factor (among others) associated with more frequent drop-in center use (Tucker et al., 2018). Rice and colleagues (Rice et al., 2022) argued that when viewed from a positive youth development perspective (Catalano et al., 2002), the benefits of drop-in centers lie in their focus on youths’ intrinsic strengths rather than deficits. In fact, positive relationships with drop-in center staff were associated with higher odds of using employment program services and greater knowledge of housing services (Rice et al., 2022). It is unclear, however, how drop in center attendance and connections to drop-in center staff are associated over time. Anecdotal conversations with providers suggest that staff perceive their relationships with youth as key facilitators of positive outcomes over time. To date, no empirical study has explored this critical association over time.

Complicating this picture is the role of information about social services and how staff may facilitate not only positive outcomes, but also may serve as critical sources of information about social services. It appears that many youth lack much information about social services available to youth experiencing homelessness, for example, surveys of high school youth in Chicago and Los Angeles indicate that youth have little knowledge or awareness of services available to runaway and homeless youth, even among those who have previously run away from home (Pergamit & Ernst, 2010). Further, lack of knowledge and other perceived barriers (e.g., too complicated, discomfort accessing services, etc.) are primary reasons why youth do not engage in higher level services, such as mental health, substance use, education, and employment services (Rabinovitz et al., 2010). It is well known that youth experiencing homelessness learn about services through their peers and support staff networks (Parast et al., 2019; Tucker et al., 2018). Therefore, relationships with staff may facilitate youths’ engagement in services by increasing their knowledge of services available to them, and their self-efficacy to use them.

## Current Study

Despite the growing body of research uncovering facilitators and barriers of service use, little research to date has investigated potential mechanisms of drop-in center service usage. To date, most research in this area has been cross-sectional at one point in time. Longitudinal research is needed to establish whether drop-in center practices are associated with subsequent positive outcomes. Statistical mediation analysis using longitudinal data can provide greater evidence for the temporality of these relationships, as well as potential causal pathways (Roth & MacKinnon, 2012). The current paper attempts to address these gaps by investigating prospective associations between positive relationships with drop-in center staff and later service use (defined as the number of different services used). Importantly, we account for youths’ frequency and duration of drop-in service use (along with other factors), as this has been significantly associated with positive staff relationships (Rice et al., 2022). We also test whether youths’ knowledge and self-efficacy as a potential mechanism of drop-in service use. We hypothesized that: (1) greater frequency and duration of drop-in center use would be associated with a greater likelihood of reporting positive relationships with staff; (2) positive relationships with staff at baseline would be directly related to subsequent service use; and (3) knowledge and beliefs about the accessibility of services would partially mediate the relationship between positive staff relationships and service use.

## Methods

### Participants and Procedures

Participants include 731 youth aged 14 to 26 (*M*_age_ = 21.8, SD = 2.2) recruited from three drop-in centers in Los Angeles, California from 2017 to 2019. Data come from a social network intervention in which peer change agents attended trainings to discuss HIV prevention behaviors with other youth at the drop-ins (Rice et al., 2021). Participants completed a baseline survey, and two follow-up surveys at 1-month (n = 476, 65.1% retained) and 3-months post-baseline (*n* = 430, 58.8% retained). All study procedures were approved by the University of Southern California Institutional Review Board.

## Measures

### Demographics

Participants self-reported their age, birth sex (“What sex were you assigned at birth, on your original birth certificate?” Male, female, or don’t know), gender identity (male, trans male/trans man, trans female/trans woman, female, genderqueer/gender non-conforming), sexual orientation (gay or lesbian, bisexual, heterosexual or straight, questioning or unsure, or asexual), and racial identity (“Pick the one that describes you best”: American Indian or Alaska Native, Asian, Black/African American, Native Hawaiian or Other Pacific Islander, White, Latino/Hispanic, and Mixed race).

### Homelessness Experiences

*Current living situation* was assessed by asking, “During the past 2 weeks, where have you spent most nights? (select one).” Participants selected from a list of eleven choices adapted from Tsemberis’ and colleagues’ (2007) Residential Time-Line Follow-back Inventory. A three-group variable was then created, indicating whether participants (1) resided in a shelter or transitional living placement, (2) were unsheltered (e.g., spent most nights in a street, park, automobile, or other location unfit for human habitation), or (3) were unstably housed (e.g., living with a family, friend, partner, i.e., “couch surfing”). Participants also indicated how long they had been homeless or unstably housed in their lifetime (with ten response options ranging from 1 = “Less than 1 month” to 10 = “9 or more years”).

### Staff Relationships

*Relationships with adult staff members* were assessed with the item, “During my time in youth services, I have developed at least one relationship with a supportive and positive staff at an agency that I attend.” Participants who responded “yes” to the prompt were considered to have a positive relationship(s) with staff, while participants who responded “No” or “Not sure” were considered not to have such a relationship.

### Knowledge and Self-Efficacy to Access Services

Participants were presented with five statements assessing their knowledge and perceived accessibility of various services, beginning with, “During my time in youth services…” Statements included: “I know who I can talk to in the youth service system to get support”, “I know what housing options are available to me”, “I have felt that drop-in services are easily accessible”, “I have felt that I know how to access shelters”, and “I have felt that I know how to connect to medical, mental health, and/or alcohol and drug support, if needed.” Responses options included 1 = “yes” and 0 = “no” or “not sure.” Responses were summed, resulting in a variable ranging from zero to five, with higher scores indicating greater perceived knowledge and accessibility of services.

### Service Use

Participants self-reported their duration (six response options ranging from 1 = “1 week or less” to 6 = “3 years +”) and frequency (seven response options ranging from 1 = “this is my first time” to 7 = “Everyday”) of drop-in center use. *Number of services used* is a count of 15 possible activities the participant engaged in “during their time in youth services” displayed as a checklist in the study survey. Meaningful activities include education and job readiness programs, art, music, counseling, pregnancy and parenting groups, etc. The full list of meaningful activities are displayed in Table 1. *Drop-in center location* is a three-category nominal variable representing where the youth received most of their services.

**Table 1:** *Endorsed Service Knowledge and Meaningful Service Engagement Items*

Variable (*n* with available data)	*n* (%)
Drop-in Center Use (*n* = 717)	
Every day	203 (28.3%)
Once or twice per week	343 (47.8%)
Once or twice per month	66 (9.2%)
Only when needed	67 (9.3%)
First time	38 (5.3%)
Relationship with Agency Staff (*n* = 711)	
At least one positive and supportive relationship	471 (66.2%)
Service Knowledge and Efficacy (1-Month Follow-up)	
I know who I can talk to in the youth service system to get support (*n* = 480)	371 (77.3%)
I know what housing options are available to me (*n =* 481)	336 (69.9%)
I have felt that drop-in services are easily accessible (*n* = 486)	394 (81.1%)
I have felt that I know how to access shelters (*n* = 481)	510 (77.5%)
I have felt that I know how to connect to medical, mental health, and/or alcohol and drug support, if needed (*n* = 486)	392 (80.7%)
Number of service knowledge items endorsed, *M (SD)* (Range: 0–5)	3.9 (1.6)
Service Engagement (3-Month Follow-up) (*n* = 430)	
Education programs (GED, college)	140 (32.6%)
Job readiness training/employment services	142 (33.0%)
Paid internship/work experience	106 (24.7%)
Job or college fair or tour	83 (19.3%)
Art or music groups	150 (34.9%)
Yoga or meditation	72 (16.7%)
Pregnancy and parenting	82 (19.1%)
Counseling and support groups	86 (20.0%)
Legal clinic	77 (17.9%)
Basic needs (shower, food, clothes, hygiene products)	269 (62.6%)
Sleep	198 (46.0%)
Case Management	205 (47.7%)
Safety	43 (10.0%)
Acceptance	50 (11.6%)
To meet / connect with non-staff	53 (12.3%)
Number of services endorsed, *M (SD)* (Range: 0–14)	2.9 (2.9)

### Data Analysis

Descriptive statistics were calculated to describe participant demographics, homelessness experiences, staff relationships, service knowledge, and service use. We first ran a preliminary regression model to test whether frequency and duration of drop-in center use at baseline was associated with reporting positive relationships with staff (cross-sectionally), accounting for demographics, living situation, peer leader status, and drop-in center location.

**Fig. 1:**
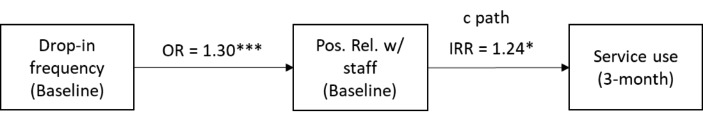
Direct Effect Path Model of Drop-in Center Service Use *Note*: All paths controlled for age, birth sex, transgender identity, LGBQ identity, non-White race, Hispanic ethnicity, shelter and unstable housing, time spent homeless (lifetime), drop-in location, and peer leader status

Next, because frequency of drop-in center use found to be the most important predictor of having a positive staff relationship at baseline (see more detailed results below), we first specified a path model in Mplus version 8 (Muthén & Muthén, 1998–2017) to test the direct effect that positive staff relationships at baseline has on service use at the 3-month follow-up (c path). In this direct effect model, frequency of drop-in center use at baseline was used to predict positive relationships with staff at baseline, and positive staff relationships at baseline was used to predict number of services used at the 3-month follow-up (see Fig. 1). Logistic regression was used to model positive relationships with staff (a binary outcome), and poisson regression was used to model number of services used at the 3-month follow-up (a count outcome). Next, we tested a model that added the indirect (i.e., mediating) effect of service knowledge, assessed at the 1-month follow-up. The indirect effect of positive staff relationships on service use (mediated through service knowledge) was tested using the Sobel method, by multiplying regression coefficients of the a path and the b path using the MODEL CONSTRAINT command in Mplus (Muthén et al., 2016). Monte Carlo integration was specified using the ANALYSIS command with the default number of integration points.

#### Missing Data Analysis

Missing data can be attributed to two causes: randomness, due to participants leaving a particular item blank, or attrition due to research staff not being able to locate individuals to complete follow-up surveys. Tables 2 and 1 display the number of cases available for univariate analysis, and Table 3 displays the number of cases available for multivariate analyses. By default, Mplus excludes cases that are missing on predictor variables in multivariate analysis. However, significant attrition did occur at follow-up (*n* = 385, 53% had missing data at follow-up). Therefore, we carried out logistic regression models to determine whether participant characteristics at baseline were significantly related to attrition at follow-up. Those missing at least one follow-up survey were more likely to be younger age (aOR = -0.09, *p* = 0.03), less likely to be peer change agents (aOR = -1.19, *p* < 0.001), attended the drop-in less frequently (aOR = -0.09, *p* = 0.04), and were more likely to have a street-based living situation, compared to participants living in a shelter (aOR = -0.72, *p* = 0.001) or who were experiencing an unstable housing situation (aOR = -0.51, *p* = 0.01). All of these variables were included as covariates in the multivariate analysis.

**Table 2:** *Participant Characteristics at Baseline Survey*

Characteristic	*M (SD)* or *n* (%)
Age, *M (SD)* (Range: 14–26) (*n* = 730)	21.8 (2.2)
Birth sex (*n* = 731)	
Male	542 (73.4%)
Female	185 (25.1%)
Don’t know	4 (0.5%)
Gender Identity (*n* = 731)	
Cisgender	638 (86.4%)
Transgender	93 (12.6%)
Race / Ethnicity (*n* = 728)	
American Indian or Alaska Native	29 (3.9%)
Asian	9 (1.2%)
Black / African American	231 (31.7%)
Native Hawaiian or Other Pacific Islander	7 (0.01%)
White	156 (21.4%)
Latino/Hispanic	112 (15.4%)
Mixed race	184 (25.3%)
LGBQ (*n* = 724)	314 (43.1%)
Living situation (*n* = 731)	
Shelter / Transitional Living Placement	170 (23.3%)
Unsheltered	304 (41.6%)
Unstably housed	257 (35.2%)

**Table 3:** *All Paths in Mediation Model Predicting Number of Services Used (N = 701)*

*Path*	*b*	*S.E.*	*p*
Pos. staff rel. ◊ Service knowledge (a path)	0.31	0.14	0.03
Service knowledge ◊ Service use (b path)	0.14	0.06	0.01
Pos. staff rel ◊ Service use (c’ path)	0.1	0.11	0.38
Indirect effect (a x b)	0.13	0.06	0.02
Total effect (c path)	0.21	0.11	0.05

*Note.* All paths controlled for age, birth sex, transgender identity, LGBQ identity, non-White race, Hispanic ethnicity, shelter and unstable housing, time spent homeless (lifetime), drop-in location, and peer leader status.

## Results

### Participant Characteristics and Service Use

As shown in Table 2, the sample was majority male sex at birth, with 12.6% being transgender and 43.1% LGBQ. Most participants were Black/African-American (31.7%), mixed race (25.3%), White/Caucasian (21.4%) or Latino/Hispanic (15.4%). 42% were unsheltered, and 23.3% were living in a shelter or transitional living placement, or were generally unstably housed (35.2%). Table 1 reports participants’ service knowledge and use of different types of services. A majority of participants used drop-ins once per week or more often, and about two-thirds reported a positive relationship with staff at baseline. A majority of participants endorsed knowledge and/or self-efficacy to use different types of services at the 1-month follow-up. Participants reported using three different types of services (*M* = 2.9, SD = 2.9) at the 3-month follow-up, with the most popular being basic needs (62.6%), case management (47.7%), sleep (46.0%), and art or music groups (34.9%).

## Path Model Results

**Fig. 2:**
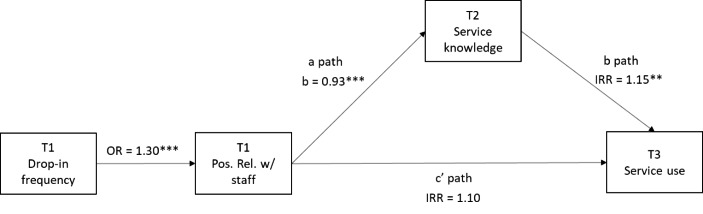
Indirect Effect Path Model of Drop-in Center Service Use *Note*: All paths controlled for age, birth sex, transgender identity, LGBQ identity, non-White race, Hispanic ethnicity, shelter and unstable housing, time spent homeless (lifetime), drop-in location, and peer leader status

Our preliminary regression model revealed that frequency of drop-in center attendance (aOR = 1.27, *p* = 0.01) was the only variable significantly associated with having a positive relationship with an adult staff member at the baseline survey (model results not shown). Next, we tested the hypothesized direct effect path model (see Fig. 1), which showed that frequency of drop-in attendance at baseline was significantly associated with positive staff relationships at baseline (aOR = 1.30, 95% CI: 1.19, 1.42), and in turn, positive staff relationships were significantly associated with number of services used at the 3-month follow-up (c path: aIRR = 1.24, 95% CI: 1.00, 1.54). Next, in the indirect effect path model (Fig. 2), greater frequency of drop-in center attendance was again associated with greater odds of reporting a positive relationship with staff at baseline (OR = 1.30, 95% CI: 1.19, 1.42). However, controlling for service knowledge at the 1-month follow-up, the association between positive staff relationships at baseline and number of services used at the 3-month follow-up was no longer significant (c’ path: IRR = 1.10, 95% CI: 0.89, 1.36). Positive staff relationship(s) with staff was significantly associated with greater service knowledge at the 1-month follow-up (a path: *b* = 0.93, *p* < 0.001). In turn, service knowledge at the 1-month follow-up was associated with a greater number of services used at the 3-month follow-up (b path: IRR = 1.15, 95% CI: 1.04, 1.28). The indirect effect was significant (*b* = 0.13, *p* = 0.02), suggesting that the association between having a positive relationship with a staff member and engaging in a greater number of services three months later was completely mediated through service knowledge.

## Discussion

There are several important findings which emerge from these results. First, the majority of the youth who were surveyed in these three drop-in centers reported having at least one positive relationship with a staff member at that agency. Prior work has also found that many youth have positive relationships with staff (Barman-Adhikari et al., 2016; Rice et al., 2011). Moreover, the majority of youth reported a sense of efficacy and knowledge about access a large number of services at the one-month follow-up survey. These services included how to find emergency shelter, how to access housing programs, how to access health and mental health services, as well as to whom to ask for help in the youth service system to gain support. In addition, at the three-month follow-up survey, youth reported using a variety of social services, consistent with cross-sectional and qualitative data reported in prior studies of youth experiencing homelessness (see Pedersen et al., 2016 for a review of this research).

Second, positive staff relationships are associated with increased service use over time. The preliminary path model displayed in Fig. 1 suggests that youth who access drop-in centers more frequently at baseline tend to report positive relationships with staff at higher rates at baseline. Then, at three months, youth who reported these positive staff relationships report using a larger number of support services. To our knowledge, this is the first time the association between positive staff relationships and service use has been demonstrated quantitatively with longitudinal data from youth accessing drop-in centers. Other studies which have relied on cross-sectional designs or qualitative data have shown the positive association between relationships to staff and behavioral health outcomes (Barman-Adhikari et al., 2016; Rice et al., 2011). Moreover, our longitudinal results are consistent with the cross-sectional results of Tucker and colleagues (2018) who found an increased sense of staff support was associated with greater drop-in service utilization. These results of the current study begin to explain the causal process of drop-in centers and youth’s relationships to staff in these centers. These data suggest that youth who come to drop-in centers more frequently are more apt to develop positive staff relationships. Over time, these positive staff relationships support youth using more services later on. It is noteworthy that our community provider authors commented that this relationship is often unacknowledged outside of youth serving organizations. Funding agencies and the general public often mis-perceive drop-in centers in largely instrumental ways, seeing them simplistically as an access point to housing and other interventions, but failing to see the critical role that relationships with staff at the centers play in the lives of the youth who use their services.

Third, and most important, these data show *how* the relationships with staff lead to service use over time. When turning to the final path model in Fig. 2, one sees that the direct path between staff relationships and service use is no longer significant, whereas the indirect path through knowledge and efficacy around services is significant. More simply put, this model suggests the following causal process: Youth who come to drop-in centers more frequently are more apt to develop positive relationships with staff. The youth who report positive relationships with staff at baseline reported a greater sense or efficacy and knowledge surrounding social services a month later. Having an increased sense of efficacy and knowledge about services at one month is then associated with an increase in different types of services used at three months. One of our community collaborating authors pointed out that this result is critical not only because it shows the causality over time, but more importantly it highlights the importance of how staff empower youth. Staff relationships enhance youth’s knowledge of services and their sense of efficacy in using those services. This knowledge and efficacy, not the staff relationship itself, is then what is associated with using more services. Our community authors have pointed out that drop-in centers are at times criticized by the general public for “enabling” youth who experience homelessness. These findings suggest that staff relationships do not enable, but rather empower youth with knowledge and efficacy which then result in youth accessing services that can lead to stability, safety and well-being.

As with any study, there are several limitations which must be acknowledged. First, we only have three months of follow-up data, and it may well be that additional insights would arise from a longer time window. Second, we have a retention rate of 60% at the 3-month follow-up, while this is similar to other studies who have follow unstable homeless youth (Bender et al., 2014). Our analyses suggest that those youth lost to follow up are different than those retained with respect to their use of drop-ins at baseline, peer change agent status associated with the parent intervention study, and living situation. This is a limitation that should be taken into account when drawing conclusions from this study, despite adjusting for these important variables in our final analysis. Third we did not examine the types of services used, but simply the number of services, so we do not know if use of particular services increased or just the variety of services. Finally, results of this study may have been influenced by social desirability bias or self-selection bias, as youth who are more engaged in services may be more likely to rate their interactions with staff positively and report greater use of services. We attempted to account for this by including youths’ frequency and duration of service use—as well as youths’ status as a peer leader in the HIV prevention intervention—as covariates in our path analysis. However, we and others argue that engagement in services should not just be viewed as a confounding variable, but a potentially important “enabling factor” in models of service use (Pedersen et al., 2016).

Perhaps the most satisfying aspect of working with community partners as co-authors are hearing their insights into future directions for research. They suggest that it is crucial for us to understand how to assist those youth who do not form positive relationships with staff. This begs the question, *How can we effectively educate this group of youth and empower them to use services?* The suggestion was made that peer models may be something to investigate. As one author stated “we discuss how our current members are the best street outreach team we could possibly have.” Exploring social network data to understand how peers impact service knowledge and use over time may prove to be very helpful. Building on our recent work utilizing peer change agents to deliver HIV prevention messaging (Rice et al., 2021), further development of peer-driven intervention models that connect youth to services may be very important (not just relying on staff relationships).

The results of this paper also point to several programming and policy implications—ideas which were generated by our service provider co-authors. First, as staff relationships are critical to youth successfully accessing a variety of other social services over time, efforts must be made to support those staff to do the work of building relationships with youth. Secondly, staff need to be compensated with salaries that encourage longer retention at youth service organizations. Third, structures need to be developed at drop-in centers that focus on the needs of staff so as to promote and encourage building ties with youth. This could include, for example, changes to the pace of work, changes to caseloads that allow for informal time with clients, or break times so that staff can be emotionally present in their interactions with clients. Fourth, funders should consider funding programs that promote relationship building between staff and clients. Such programs may not appear instrumental, but staff relationships empower youth with knowledge and self-efficacy over time and this empowerment leads to the use of a wide array of services that promote long term stability and well-being.
